# Radiomics-guided deep neural networks stratify lung adenocarcinoma prognosis from CT scans

**DOI:** 10.1038/s42003-021-02814-7

**Published:** 2021-11-12

**Authors:** Hwan-ho Cho, Ho Yun Lee, Eunjin Kim, Geewon Lee, Jonghoon Kim, Junmo Kwon, Hyunjin Park

**Affiliations:** 1 Department of Electrical and Computer Engineering, Sungkyunkwan University, Suwon, Republic of Korea; 2 Center for Neuroscience Imaging Research, Institute for Basic Science, Suwon, Republic of Korea; 3 Department of Radiology and Center for Imaging Science, Samsung Medical Center, Sungkyunkwan University School of Medicine, Seoul, Republic of Korea; 4 Department of Health Sciences and Technology, Samsung Advanced Institute for Health Sciences & Technology (SAIHST), Sungkyunkwan University, Seoul, Republic of Korea; 5 Department of Radiology and Medical Research Institute, Pusan National University Hospital, Pusan National University School of Medicine, Busan, Republic of Korea; 6 School of Electronic and Electrical Engineering, Sungkyunkwan University, Suwon, Republic of Korea

**Keywords:** Prognostic markers, Lung cancer

## Abstract

Deep learning (DL) is a breakthrough technology for medical imaging with high sample size requirements and interpretability issues. Using a pretrained DL model through a radiomics-guided approach, we propose a methodology for stratifying the prognosis of lung adenocarcinomas based on pretreatment CT. Our approach allows us to apply DL with smaller sample size requirements and enhanced interpretability. Baseline radiomics and DL models for the prognosis of lung adenocarcinomas were developed and tested using local (*n* = 617) cohort. The DL models were further tested in an external validation (*n* = 70) cohort. The local cohort was divided into training and test cohorts. A radiomics risk score (RRS) was developed using Cox-LASSO. Three pretrained DL networks derived from natural images were used to extract the DL features. The features were further guided using radiomics by retaining those DL features whose correlations with the radiomics features were high and Bonferroni-corrected p-values were low. The retained DL features were subject to a Cox-LASSO when constructing DL risk scores (DRS). The risk groups stratified by the RRS and DRS showed a significant difference in training, testing, and validation cohorts. The DL features were interpreted using existing radiomics features, and the texture features explained the DL features well.

## Abstract (web summary)

Cho et al. use a radiomics-guided deep-learning approach to model the prognosis of lung adenocarcinoma from CT scan data. This study demonstrates the utility of this technology as a predictive approach for stratifying clinical prognostic groups.

## Introduction

Lung adenocarcinoma is one of the most common cancers, and because it can lead to vastly different clinical outcomes, a proper stratification of its prognosis is critical^1^. Lung adenocarcinoma is divided into low, intermediate, and high-grade prognostic groups according to the most predominant pattern evaluated by histopathology, not in vivo imaging^1–4^. However, even among lung adenocarcinomas with the same most predominant pattern, the spectrum of actual prognosis varies widely^3,5–7^. The presence of any high-grade pattern such as a micropapillary and solid pattern is known to have a poor prognosis regardless of the predominant pattern^6,8,9^. A proper stratification of prognosis for operable lung cancer allows us to prevent local treatment failure by finding high-risk patients who cannot be fully treated with standard management^10–13^. It will lead to pursuing more aggressive treatments such as adjuvant treatment or open thoracotomy for those high-risk patients. Imaging, particularly computed tomography (CT), has been widely used for the prognosis of lung adenocarcinoma using semantic features, such as shape, size, and margin derived from expert knowledge. Recent studies adopted approaches based on radiomics to assess the risk of various types of cancer^14,15^. In particular, the efficacy of radiomics analysis on lung cancer using routine clinical imaging such as CT and PET has been demonstrated in many studies^16–21^. Some studies successfully showed that prognostic models based on radiomics are feasible in lung cancer^22–25^. In radiomics, imaging data are transformed into high-dimensional features, and tumors are quantified with such features typically focusing on intra-tumoral heterogeneity or tumor appearance^26–31^. Radiomics can effectively decode tumor properties associated with treatment response, prognosis, and diagnosis^26–31^. Furthermore, radiogenomics, a method for integrating two types of high-dimensional information, one from imaging and the other from genomics, can lead to a comprehensive interpretation of the tumor^32–34^. Radiomics mostly adopts features that are mathematically or semantically defined and hence are referred to as handcrafted features based on expert knowledge. Even if many radiomics features are present, abstract information from imaging data that are not properly modeled using our current semantic knowledge could remain uncovered^14,35^.

A branch of artificial intelligence known as deep learning (DL) has recently made a meaningful impact on medical imaging, propelled by advances in computing hardware, algorithms, and big data^36^. In non-medical imaging fields using natural images, the performance of DL algorithms has routinely exceeded that of humans^37,38^. Such advances are less common in the medical imaging field because the number of imaging data and the associated annotations are far scarcer than those in natural images^39^. One way to circumvent the issue of the sample size is to adopt DL networks pretrained from natural images^40,41^. The burden of the sample size is decreased because we can simply apply or fine-tune the pretrained model using fewer samples as compared to developing a DL network from scratch. Especially, DL networks trained to recognize objects in natural images can be effectively applied to medical imaging because abstract high-level information is common between natural and medical images^42^. Another major drawback of DL is the difficulty of interpretation. DL studies on lung cancer offered limited explanations using gradient-weighted class activation mapping^43–46^. The approaches highlight important regions contributing to the DL network and have a weak link to the underlying biology. This issue becomes more critical in medical imaging because the decisions of the algorithms require well-rooted explanations for use in clinical practice^47^. Features derived from DL models are difficult to interpret and linking them to features that are more interpretable such as handcrafted radiomics features could enhance the interpretability of the DL models.

In this study, we aimed to propose a methodology that applies pretrained DL models in a radiomics-guided manner and to verify the proposed method by stratifying the prognosis of lung adenocarcinoma. We hypothesize that the use of the pretrained DL model and linkage with radiomics features allow us to explore DL-derived features with smaller sample size requirements and enhanced interpretability. A prognostic model was built using the proposed approach for the training cohort and further validated in an external cohort.

## Results

### Overall workflow

**Fig. 1:**
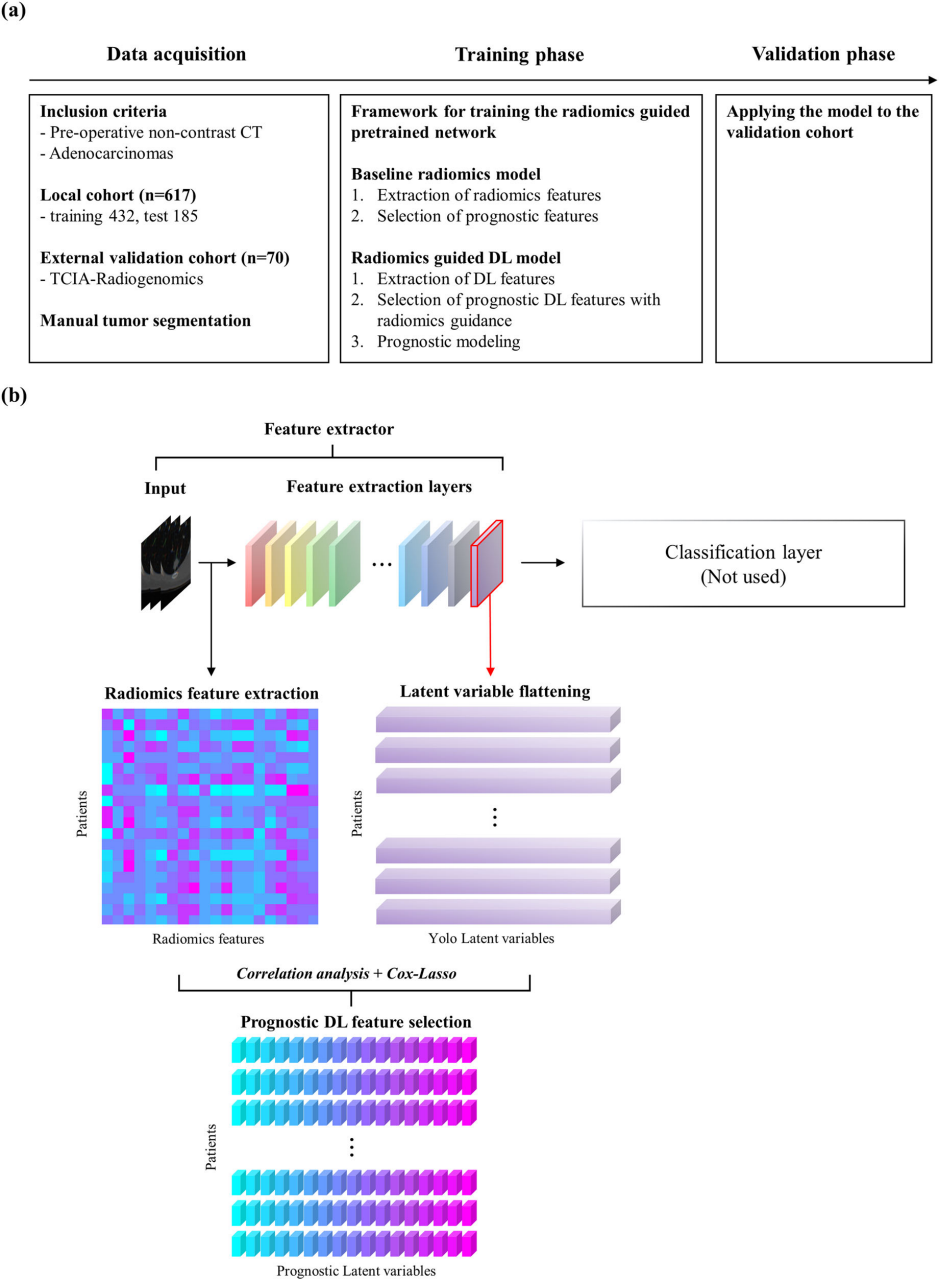
Overall workflow of the study. (**a**) Three major procedures used in this study. (**b**) Graphical description of DL feature selection guided by radiomics used for prognosis.

The overall workflow is shown in Fig. 1. We studied 617 patients from a local cohort and 70 patients from an external validation cohort in the public domain with lung adenocarcinoma. Preoperative CT images were analyzed and survival analysis was performed based on Cox’s proportional hazard model with a few features. As a baseline, we computed radiomics features and built a radiomics risk score (RRS). Three pretrained DL networks were used as feature extractors and the extracted DL features were subjected to correlation analysis with radiomics features to retain important radiomics-guided features. The selected features were used to build a DL risk score (DRS) for each network. The DRS models were compared with RRS and important DL features were interpreted with radiomics features. Full details are provided in the “Methods”.

### Baseline radiomics model for prognosis

The local cohort (*n* = 617) was randomly divided into a 7:3 ratio and used as training and test cohorts. ROI reproducibility in terms of Cohen’s kappa was a mean (standard deviation [SD]) of 0.8916 (0.0416), whereas feature reproducibility of the radiomics in terms of intra-class correlation coefficient (ICC) of all features was a mean (SD) of 0.9533 (0.0563). More details on the ICCs are in Supplementary Data 1. Table 1 describes the RRS model for prognosis using the selected features. The RRS model was built using one shape feature, three texture features, and one margin feature. The shape feature was the maximum 2D diameter associated with T stage^48^. Two of the texture features were from the gray-level co-occurrence matrix (GLCM), and the other was from the gray-level size-zone matrix (GLSZM). Autocorrelation of GLCM measures the fineness and coarseness of texture, the informational measure of the correlation of GLCM quantifies the complexity of intra-tumoral heterogeneity, and large area emphasis of GLSZM measures the distribution of large-sized clusters whose high value indicates a greater coarseness in texture^49^. The marginal feature was the skewness of the cumulative distribution function (CDF) slope reflecting the degree of pathological invasiveness of tumor^50^.

**Table 1:** Radiomics risk score model for prognosis and the associated radiomics features.

Category	Feature	Cox-LASSO coefficient
Shape	Maximum 2D dameter	0.1581
GLCM	Autocorrelation	0.0409
GLCM	IMC2	−0.0050
GLSZM	Large area emphasis	−0.0067
Margin	CDF slope skewness	0.1930

*GLCM* gray-level co-occurrence matrix, *GLSZM* gray-level size-zone matrix, *IMC* informational measure of correlation, *CDF* cumulative distribution function. The optimal penalty is the penalty term of the Cox-LASSO model.

**Fig. 2:**
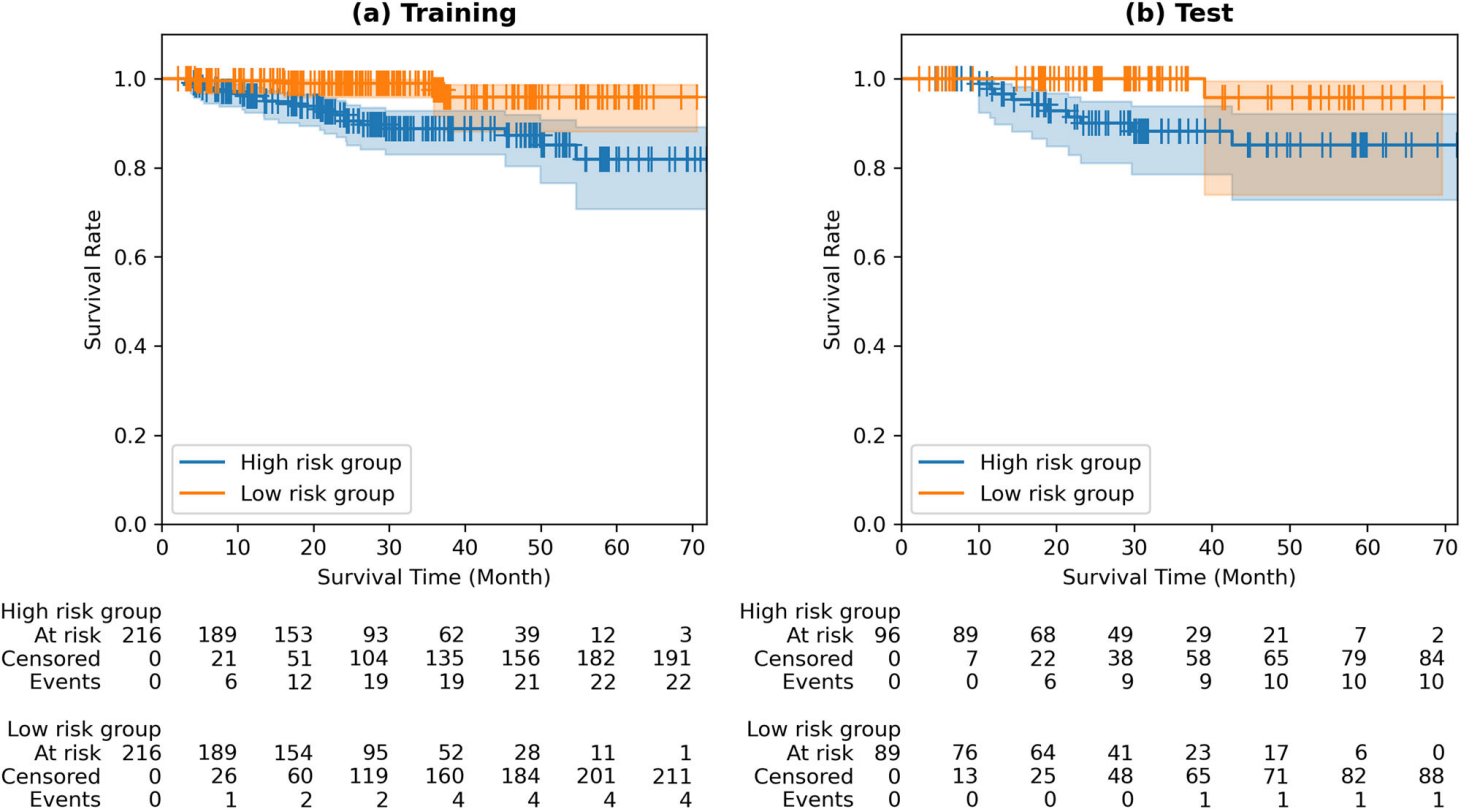
Kaplan–Meier plots of the baseline radiomics model. **a** Kaplan–Meier plot of training cohort, hazard ratio of 4.1384 (95% CI of 1.9140–8.9479), *p*-value of 0.0006, C-index of 0.7459. **b** Kaplan–Meier plot of test cohort, hazard ratio of 5.0566 (95% CI of 1.5379–16.6263), *p*-value of 0.0180, and C-index of 0.6837.

Figure 2 shows a Kaplan–Meier plot using RRS for both training and test cohorts. Survival analysis using baseline radiomics was limited to the training and test cohorts where the ROI was available. The risk group stratified by the RRS showed a significant difference in both training and test cohorts. We observed a hazard ratio (HR) of 4.1384 (95% confidence interval (CI) 1.1940–8.9479), *p*-value of 0.0006, and Concordance index (C-index) of 0.7459 for the training cohort, whereas an HR of 5.0566 (95% CI 1.5379–16.6263), *p*-value of 0.0180, and C-index of 0.6837 were observed for the test cohort. Patients with high-risk scores were candidates for close surveillance or adjuvant therapy even in an early stage. The same criterion was applied to both the local and validation cohorts.

### Radiomics-guided pretrained DL model for prognosis

Through the first stage of prognostic DL feature filtering, we retained 30, 51, and 90 DL features with corresponding Bonferroni-corrected *p*-values of 3.2 × 10^−13^, 5.9 × 10^−13^, and 1.2 × 10^−13^ for Yolo, DenseNet, and VGG, respectively. After the Cox-LASSO feature selection, three, five, and five features remained and were used to build DRS models for Yolo, DenseNet, and VGG, respectively. The performance of various radiomics-guided DL models is shown in Table 2.

**Table 2:** Performance of various radiomics-guided DL models.

	Training		
	Hazard ratio (95% CI)	*p*-value	C-index
Radiomics	4.1384 (1.9140–8.9479)	0.0006	0.7459
Yolo	3.1728 (1.4675–6.8596)	0.0061	0.7994
DenseNet	4.4383 (2.0468–9.6241)	0.0003	0.7410
VGG	4.2454 (1.9658–9.1683)	0.0005	0.7660
	**Test**		
	**Hazard ratio (95% CI)**	***p*-value**	**C-index**
Radiomics	5.0566 (1.5379–16.6263)	0.0180	0.6837
Yolo	7.6277 (2.2870–25.4399)	0.0027	0.7593
DenseNet	5.3620 (1.7011–18.5774)	0.0116	0.7214
VGG	4.7060 (1.4333–15.4520)	0.0244	0.8009
	**Validation**		
	**Hazard ratio (95% CI)**	***p*-value**	**C-index**
Radiomics	–	–	–
Yolo	6.5362 (2.1773–19.6213)	0.0022	0.7696
DenseNet	4.2277 (1.4800–12.0768)	0.0153	0.7112
VGG	3.5488 (1.2409–10.1494)	0.0362	0.6757

**Fig. 3:**
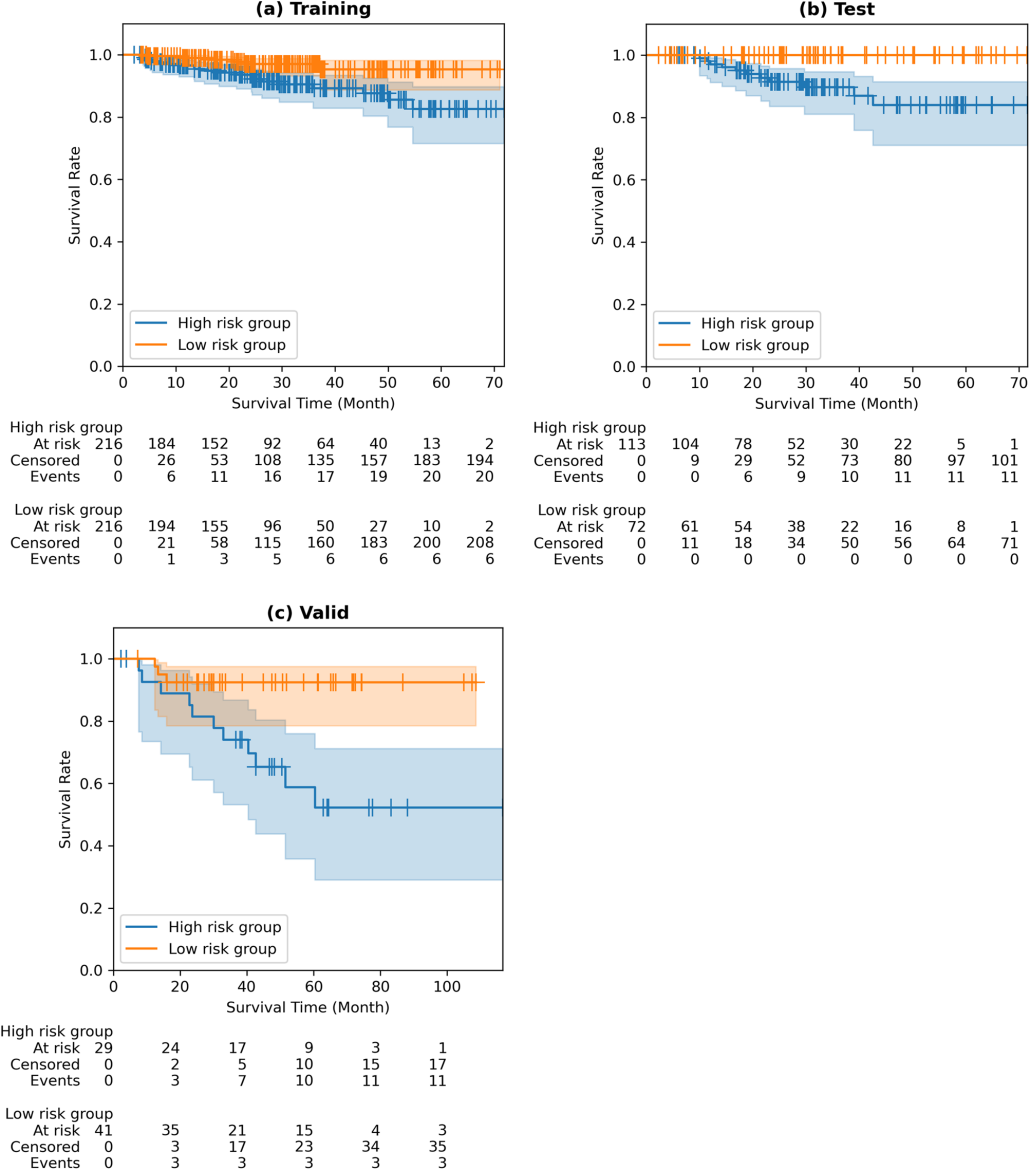
Kaplan–Meier plots of radiomics-guided DL (Yolo) model. **a** Kaplan–Meier plot of training cohort, hazard ratio of 3.1728 (95% CI of 1.4675–6.8596), *p*-value of 0.0061, and C-index of 0.7994. **b** Kaplan–Meier plot of test cohort, hazard ratio of 7.6277 (95% CI of 2.2870–25.4399), *p*-value of 0.0027, and C-index of 0.7593. **c** Kaplan–Meier plot of validation cohort, hazard ratio of 6.5362 (95% CI of 2.1773–19.6213), *p*-value of 0.0022, and C-index of 0.7696.

Table 3 describes the best DRS model (i.e., Yolo model) for prognosis using the selected DL features, and Fig. 3 shows the Kaplan–Meier plot using the DRS model for the training, test, and validation cohorts. The risk group stratified by the DRS showed a significant difference in all cohorts. We observed an HR 3.1728 (CI 1.4675–6.8596), *p*-value 0.0061, and C-index 0.7994 in the training cohort, whereas an HR 7.6277 (CI 2.2870–25.4399), *p*-value 0.0027, and C-index 0.7593 were observed in the test cohort. In the external validation cohort (TCIA-Radiogenomics), an HR 6.5362 (CI 2.1773–19.6213), *p*-value 0.0022, and C-index 0.7696 were observed. The risk stratification using the DRS in the training and test cohorts was better than that using the RRS in terms of the C-index, demonstrating the effectiveness of the DL approach. DRS models using Dense and VGG networks are shown in the supplementary document (Figs. S1, S2; Tables S1, S2). Histogram plots of RRS/DRS for both risk groups are given with median cut-off (Fig. S3).

**Table 3:** DL (Yolo) risk score model for prognosis and the associated DL features.

Feature	Cox-LASSO coefficient
Yolo_Latent_1145	−0.0376
Yolo_Latent_1161	−0.0632
Yolo_Latent_22664	0.5524

The numbers appearing as the postfix of the feature names denote the index of the DL features. The optimal penalty is the penalty term of the Cox-LASSO model.

### Interpretation of the DL features with radiomics

During the filtering procedure to identify the potential prognostic DL features, we computed correlations of all possible pairs of DL and radiomics features. This allows us to interpret the selected DL features in the context of radiomics features. Table 4 shows the radiomics features associated with prognostic DL features and their correlation. Yolo_Latent_1145 feature was significantly (*p* < 0.05) correlated with 60 radiomics features. The numbers appearing as the postfix of the feature names denote the index of DL features. Among them, 14 radiomics features showed an absolute correlation of over 0.4, and the inverse difference normalized feature of GLCM was the most significantly correlated (*r* = −0.5057). Yolo_Latent_1161 was significantly correlated with 63 of the radiomics features, 17 of the radiomics features had an absolute correlation of over 0.4, and the inverse difference of GLCM was the most significantly correlated (*r* = −0.5743). In addition, Yolo_Latent_22664 was significantly correlated with 54 radiomics features. Only one radiomics feature showed an absolute correlation value of over 0.4 and the inverse difference of GLCM was the most significantly correlated (*r* = 0.4009).

**Table 4:** DL (Yolo) risk score model for prognosis and the associated DL features.

Prognostic DL feature	Category	Radiomics feature	Correlation
Yolo_Latent_1145			
	Histogram	Entropy	0.4559
	Histogram	Uniformity	−0.4188
	GLCM	Difference average	0.481
	GLCM	Difference entropy	0.493
	GLCM	Inverse difference	−0.5057
	GLCM	IDM	−0.4893
	GLCM	IDMN	−0.4025
	GLCM	IDN	−0.4966
	GLCM	IMC2	0.4124
	GLCM	Inverse variance	−0.493
	GLCM	Sum entropy	0.4148
	GLSZM	Zone percentage	0.4718
	Margin	CDF slope skewness	−0.4467
	Margin	CDF slope kurtosis	−0.432
Yolo_Latent_1161			
	Shape	Maximum 2D diameter	−0.4104
	Histogram	Entropy	0.5011
	Histogram	Uniformity	−0.4517
	GLCM	Contrast	0.4523
	GLCM	Difference average	0.5585
	GLCM	Difference entropy	0.5581
	GLCM	Inverse difference	−0.5742
	GLCM	IDM	−0.5538
	GLCM	IDMN	−0.4748
	GLCM	IDN	−0.5743
	GLCM	IMC1	−0.4199
	GLCM	IMC2	0.4441
	GLCM	Inverse variance	−0.5601
	GLCM	Sum entropy	0.4551
	GLSZM	Zone percentage	0.5417
	Margin	CDF slope skewness	−0.4905
	Margin	CDF slope kurtosis	−0.4665
Yolo_Latent_22664			
	GLCM	IDN	0.4009

*GLCM* gray-level co-occurrence matrix, *GLSZM* gray-level size-zone matrix, *IMC* informational measure of correlation.

The first two DL features correlated with many handcrafted radiomics features and thus we could interpret them as combined features from the interpretable radiomics features. The first DL feature, Yolo_Latent_1145, could be interpreted as a combination of two histogram features, 10 texture features, and two marginal features. All 14 features have mathematical definitions and hence offer some degree of explanation. Especially, the texture features are biologically rooted and offer a potential explanation of how they are important. The inverse difference feature of GLCM measures local homogeneity and was the most contributing radiomics feature for Yolo_Latent_1145. The second DL feature, Yolo_Latent_1161, can be interpreted as a combination of one shape feature, two histogram features, 12 texture features, and two marginal features. The largest portion (12 out of 17 features) was from the texture feature similar to the first DL feature confirming the importance of the texture features. The inverse difference normalized feature of GLCM also measures local homogeneity and was the most contributing radiomics feature for Yolo_Latent_1161. The first two DL features (Yolo_Latent_1145 and Yolo_Latent_1161) have many common radiomics features and most of the common ones belong to the texture features. The texture features mostly reflect tumor heterogeneity and thus can be biologically interpreted. In short, two prognostic DL features largely depend on radiomics texture features related to the heterogeneity of the tumor. The third DL feature, Yolo_Latent_22664, could be interpreted as a surrogate for one texture feature, again confirming the importance of the texture features. Yolo_latent_1145 and Yolo_Latent_1161 have negative contributions towards high risk (i.e., negative Cox-LASSO coefficients), while Yolo_Latent_22664 has a positive contribution towards high risk. This implies that the most related radiomics feature such as the inverse difference normalized feature of GLCM could have an opposite direction of correlation.

**Fig. 4:**
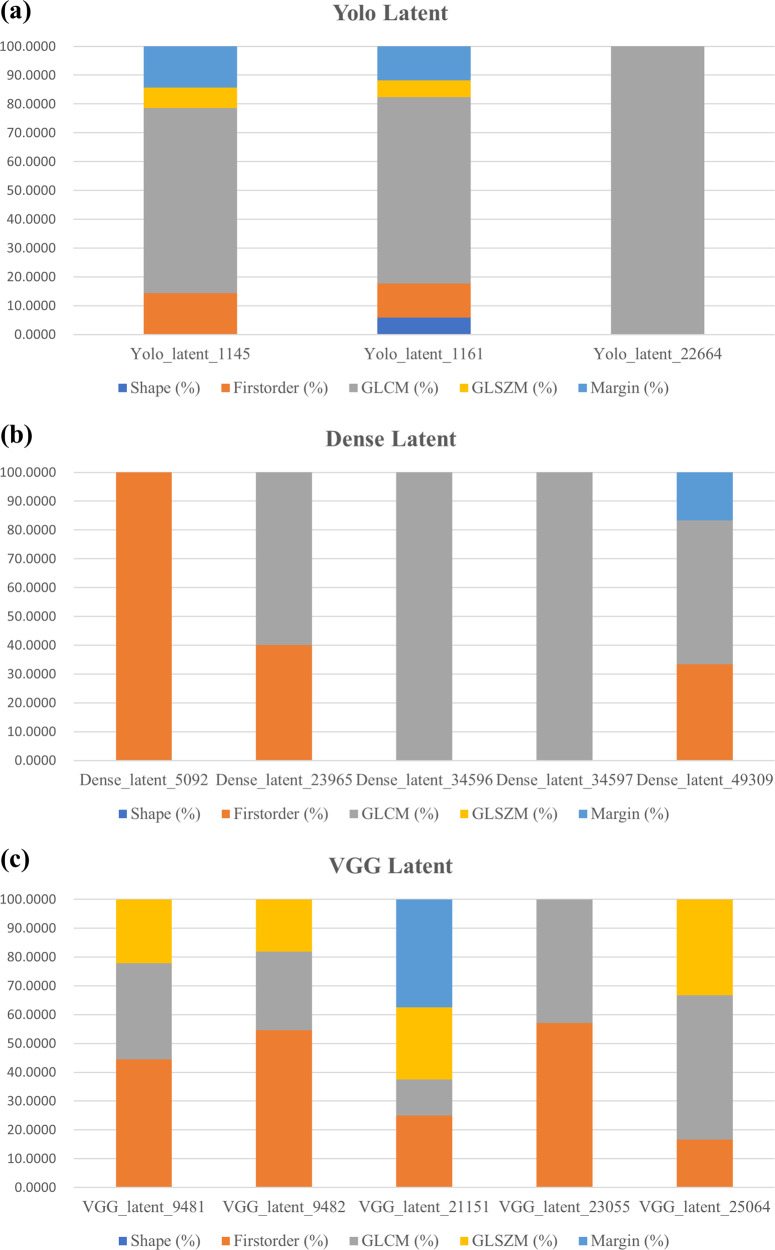
Comparison of different proportions of radiomics features in five categories associated with the prognostic DL features for the three networks. The five categories are shape (in blue), first-order (in orange), GLCM (in gray), GLSZM (in yellow), and margin (in light blue) categories. **a** Yolo network. **b** DenseNet. **c** VGG.

Figure 4 shows how each prognostic DL latent feature has different proportion of associated radiomics features in five categories for the three networks. Three DL networks provided different features and after the Cox-LASSO procedure, different sets of features were selected. Only the selected prognostic features for each network were interpreted with radiomics features in Fig. 4. The texture information (i.e., GLCM, GLSZM) was dominant in all latent DL features. Histogram-based features and margin features were also found in some latent DL features. Shape features were the most scarce.

## Discussion

In this study, we proposed a methodology using a pretrained DL model in a radiomics-guided manner and found that our method can explore DL features for the prognostication of lung adenocarcinomas, requiring a relatively smaller sample size and enhancing interpretability. The radiomics-guided approach (i.e., DRS) outperformed the baseline radiomics approach (i.e., RRS) demonstrating the effectiveness of the DL approach evaluated in the training, test, and validation cohorts.

Proper prognosis stratification for operable lung cancer is important because treatment options for lung adenocarcinoma vary depending on the situation. For example, adenocarcinoma with non-predominant micropapillary or solid component shows aggressive behavior and may require adjuvant treatment after resection^13^. Early lung cancers with nodal metastasis may not be completely operated by the widely used video-assisted thoracic surgery, and thus require open thoracotomy^10–12^. In sum, prognosis stratification allows identifying high-risk patients that are difficult to manage using conventional operations. Our results show that prognosis stratification using preoperative imaging is feasible by radiomics-guided DL approach.

In our study, the radiomics part is necessary for two aspects. First, its main role is to guide the selection of DL features. We retained DL features that showed significant correlations with radiomics features that are interpretable. Our approach adopted radiomics-guided DL features and thus is more interpretable than using many DL features without selection. However, using many DL features without radiomics guidance might lead to a better survival prediction that is more difficult to interpret. Second, the radiomics part serves as a baseline model for comparison. Radiomics is a well-established analysis method using conventional machine learning approaches and thus is a suitable choice for comparison.

We observed the performances of our DL models with respect to the different number of training samples in 100 sample increments for the test and validation cohorts (Table 5). There were no statistical differences between the subsampled and the whole training sets for survival and death events. The same model construction procedures were applied with fewer samples. The C-index gradually increased as we employed more samples and saturated after using 300 samples. The HR and *p*-value improved as more samples were used as well. In particular, a significant *p*-value was obtained for 200+ samples in the test cohort and a significant *p*-value was obtained for 300+ samples in the validation cohort. These results might imply that a training sample of 300 or more could lead to sufficient model performance but additional performance gain might be possible as we increase the training sample size.

**Table 5:** Performance of DL (Yolo) risk model with respect to the different number of training samples for the training and validation cohorts.

	Test			
Number of samples	Hazard ratio (95% CI)		*p*-value	C-index
Whole (*n* = 432)	7.6277 (2.2870–25.4399)		0.0027	0.7593
300	6.4256 (1.9081–21.6386)		0.0071	0.7950
200	4.0644 (1.2211–13.5284)		0.0478	0.6368
100	2.6913 (0.8064–8.9821)		0.1927	0.5988
	**Validation**			
**Number of samples**	**Hazard ratio (95% CI)**		***p*-value**	**C-index**
Whole (*n* = 432)	6.5362 (2.1773–19.6213)		0.0022	0.7696
300	3.9521 (1.3726–11.3794)		0.0228	0.7724
200	2.4826 (0.8542–7.2149)		0.1620	0.5135
100	2.2463 (0.8442–7.1475)		0.1686	0.5391

Our DL model adopted a 2.5D model, which considered three consecutive axial slices centered at the tumor centroid, not a full 3D model where a full 3D CT spanning tens of slices is considered. Models based on the volumetric convolutions (i.e., 3D models) require high computational power and memory owing to a large number of parameters^39–42^. Because our model uses 2.5D with only a few slices to consider, it has a lighter computational burden. Still, 3D models can be more precise and provide a comprehensive abstraction based on the information from the entire 3D volume, although this requires a high sample size, which limits their usage in medical imaging. One of our motivations was to tackle the issue of the sample size, and thus the use of the 2.5D model can be effective.

The DL features were derived from three well-known DL networks with frozen weights (i.e., no optimization). Since we are not finetuning the weights, we do not need many samples. Correlation analysis between 78 radiomics features and tens of thousands (or hundreds of thousands) features is performed to filter prognostic DL features guided by radiomics. The correlation analysis requires fewer samples than retraining a large capacity (million or more weights) DL model. Thus, we believe our approach contributes towards using fewer samples.

An interpretable model with a small sample size requirement was possible through the proposed method, still, the wide CI of HR may imply that the training sample size is not sufficient in general. Our sample size of 617 for the local cohort might be on the small side for natural images, but for medical imaging studies, it is a fair number. We are not optimizing millions of weights as is done in DL training. Instead, we are only optimizing a few parameters as is typically done in conventional machine learning studies. Existing radiomics and medical AI studies recommended sample size to be at least five to ten times the selected features^51^. Our representative DL risk model using Yolo have three features and we have more than 100 samples per selected feature (local cohort of *n* = 617 split into a train set of *n* = 432 and a test set of *n* = 185). Still, the CI of the proposed model might decrease with more samples.

We extracted latent variables (i.e., deep learning features) from the last layer of each pretrained network. Previous studies used the features of the layer, but they typically combined or concatenated the features with the radiomics feature without selecting prognostic features^52–54^. This leads to a lack of interpretability. Moreover, the last layer tends to contain the most abstract information and thus we believe the features from the last layer would be good candidates to correlate with radiomics features.

We used the last layer because it contains the most abstract information. Still, important features could be present in the intermediate layers. Performance of various radiomics-guided DL models using features extracted from intermediate layers is reported in the Supplement (Table S3). Our intention is not to interpret the entire prediction process of DL networks. We retained DL features correlated with radiomics in the final layer, not all layers so that the few selected DL features become interpretable. Since we chose a few correlated DL features, the ensuing Cox model is a compact model that is less susceptible to overfitting compared to using many DL features.

During the step used to filter the potential prognostic DL features correlated with radiomics features, we applied a threshold of 0.4 for the correlation value, which led to retaining the top 5% associated features. We explored using other thresholds, and a threshold value of 0.3 led to retaining 263 radiomics-associated DL features, where the related DL model showed an HR 2.7019 (CI 1.2494–5.8432) with *p*-value 0.0197 for the training cohort, an HR 7.6754 (CI 2.3150–25.4472) with *p*-value 0.0025 for the test cohort, and an HR 4.0050 (CI 1.8806–15.6616) with *p*-value 0.0213 for the validation cohort. The threshold value of 0.5 led to retaining seven radiomics-associated DL features, and the related DL model showed an HR 3.2050 (CI 1.1413–6.9347) with *p*-value 0.0058 for the training cohort, an HR 7.2715 (CI 2.1746–24.3151) with *p*-value 0.0036 for the test cohort, and an HR 5.4270 (CI 1.8806–15.6616) with *p*-value 0.0043 for the validation cohort. Using threshold values of 0.3 and 0.5 brought about lower HRs for the validation cohort than using the threshold value of 0.4. A higher threshold (>0.5) led to no retaining of any of the DL features. A lower threshold (<0.3) made the correlation too low, which made the association with the radiomics features less meaningful. As a result, we chose a threshold value of 0.4, which retained the top 5% of the DL features.

Many studies successfully transferred pretrained DL networks trained from natural images to medical imaging^55^. Various tasks including tumor segmentation and response prediction were possible in many different organs and imaging modalities. This is mainly because the pretrained DL models rely heavily on convolutional neural networks that can extract a high-level abstraction from images. Such abstractions are common between natural and medical images in contrast to low-level information (i.e., pixel intensity information) that is potentially different between the two. Our study also confirmed that transferring the pretrained DL networks was effective for analyzing lung adenocarcinoma.

In our baseline radiomics model, one shape feature, three texture features, and one margin feature were identified in the RRS (Table 1). Maximum 2D diameter, a shape feature, is well known to be directly associated with the T stage reflecting the tumor burden^48^. A higher maximum 2D diameter implied higher RRS in our model. This was rather expected as larger tumors tend to show a poor prognosis. Intra-tumoral coarseness and the complexity of texture are also important in an RRS. In detail, autocorrelation of GLCM measures the degree of linear dependency of elements in GLCM, informational measure of correlation of GLCM reflects texture complexity correlated with the intra-tumoral genomic heterogeneity, and large area emphasis of GLSZM measures the distribution of large-sized clusters whose high value indicate greater coarseness in texture, reflecting a poorer differentiation in histopathology^49^. Finally, the skewness of the CDF slope, the margin feature, is associated with the degree of invasiveness in tumor and manifests the interaction between the tumor and surrounding lung parenchyma^50^. In well-defined tumors, negative skewness in CDF is likely due to a rapid change in CDF. Higher skewness of the CDF slope led to a higher RRS in our model, which implies that the prognosis is poor with ill-defined tumors.

In the DRS model, we observed three prognostic DL features (Table 4). Yolo_Latent_1145, Yolo_Latent_1161, and Yolo_Latent_22664 showed absolute correlation over 0.4 with 14, 17, and one radiomics features, respectively. Each prognostic DL feature is explainable as the combination of different handcrafted radiomics features. Radiomics features reflecting texture were the most dominant in all three DL features confirming the importance of texture in prognosis. Furthermore, the shape and margin features, maximum 2D diameter and skewness of the CDF slope, which were included in the RRS, were also reported as correlated with the DL features. The prognostic DL features are multifaceted reflecting texture, shape, and margin, and thus can be considered novel abstract features obtainable through DL approaches. The multifaceted nature of the DL feature might be the reason behind the improved performance of the DRS as compared to RRS.

A recent study applied a shallow 3D convolutional neural network to predict the prognosis of lung cancer patients using multi-institutional CT datasets^56^. To the best of our knowledge, this is the state-of-the-art DL model for lung adenocarcinoma prognostication. This study also adopted a transfer-learning framework to build the prognostic model noted as LungNet. They used five datasets (*n* = 307) and each dataset included data from up to ten sites. Although multiple open-source and local datasets were used to conduct the study, and non-imaging clinical features were added, their prognostic performance was worse (C-index 0.74) than ours for the same dataset in our external validation cohort (i.e., TCIA-Radiogenomics). Our model did not consider clinical features and was trained using fewer imaging data but showed better prognostic performance (C-index 0.77) for the external validation cohort. One possible reason could be that a simple 2.5D model is better suited than a more complex 3D model with limited samples and guidance with radiomics features retaining only informative DL features relevant to prognosis.

There are several limitations to our study. Our model adopted a 2.5D model with a less computational burden. The 3D models can be more precise and provide comprehensive abstraction from the whole 3D volume, which requires further study. The demographic information between the local and external cohorts showed significant differences in many aspects. This is partly due to the healthcare environment in South Korea where routine lung cancer screening is widely applied enabling early diagnosis leading to a better prognosis. We used a non-contrast CT for our study, although there were imaging acquisition-related differences between the two cohorts. The manufacturer and image acquisition parameters were not strictly controlled. This is mainly due to the inherent constraints of the open-source datasets. Contrast CT from the open-source dataset bears additional variations owing to the difference in how the contrast agents are administered, whereas non-contrast CT has the advantage of reflecting the relationship between HU and tumor density/cellularity. Thus, we chose the non-contrast CT. Future prospective studies controlling for the imaging parameters are necessary to fully validate the findings of our study. Label errors are widespread and the medical field is no exception^57^. Our model predicted survival information that contains censored data. Thus, not all death events were modeled due to missing follow-up. Certain radiomics features appeared in common analyzing the prognostic DL features. They are good candidates to forcefully add to the RRS. However, this makes the comparison among models unfair because all our models were data-driven ones. Thus, we plan to explore this option to incorporate important handcrafted features in the future. Finally, our DL features were explained in terms of handcrafted radiomics features. All radiomics features are defined mathematically and hence interpretable in that sense; however, only a fraction of them have explicit biological explanations. Such explanations are more difficult for lung cancers because they are surrounded by air, unlike many types of tumors in solid organs. Thus, the explanations should consider the specific microenvironment of lung cancers. Further studies exploring the biological rationale of radiomics features, possibly in the form of radiogenomics, are necessary to advance this field.

Deep learning models can uncover high-level abstract information for raw imaging data, but suffer from an increased sample size and interpretability issues. Our approach using a pretrained DL network in a radiomics-guided manner allowed us to explore DL features with smaller sample size requirements and enhanced interpretability. Our approach was only tested in the context of lung adenocarcinoma, although it can be easily extended to other cancer types in different organs. We adopted three representative pretrained networks to extract DL features, but other pretrained networks can be easily used as well.

In future work, studies using more samples are needed to see if we can reduce the CI of the hazard ratio for more robust results. We also need to adjust and fine-tune existing networks. Training a new network with additional layers and interpreting the final layer of the new network might lead to better performance. This may lead to more efficient prognostic task-specific modeling. Finally, this study focused on the interpretation of the latent variables of the last layer. A more in-depth study of the intermediate layers might lead to a comprehensive understanding of the prediction processes of deep learning networks.

## Methods

### Patient cohorts and imaging collection

For the training and test cohorts, this single-center prospective study was approved by the institutional review board of Samsung Medical Center (SMC 2011-09-083), and informed consent was obtained from all patients. This study was conducted as a part of an ongoing prospective clinical trial of early stage lung adenocarcinoma patients who underwent preoperative CT scans (NCT01482585) from January 2014 to July 2019 at Samsung Medical Center (Seoul, Korea). We included patients who were scheduled to undergo curative surgery. Patients with a history of previous radiation or chemotherapy were excluded. The training cohort was used to identify a few DL features correlated with radiomics features and compute coefficients of the prognostic model in the selected DL features.

For the local cohort, CT images were obtained using a CT scanner (Somatom Definition Flash; Siemens Healthcare, Forchheim, Germany) with the following parameters: detector collimation of 0.625 mm, 120 kVp, 150–200 mA, and a reconstruction interval 1 mm. Image data were reconstructed using a soft-tissue algorithm for mediastinal window ranges and a bone algorithm for lung window images. Table 6 shows the demographic information of the study cohorts.

**Table 6:** Demographic information of study cohorts.

		Local (train/test)	Validation	*p*-value
*n*		617	70	
Age	Mean (STD)	60.2172 (9.3683)	66.9714 (11.1797)	<0.0001
Sex				0.0443
	Male	266	39	
	Female	351	31	
Smoking history				<0.0001
	No	384	20	
	Yes	233	50	
Follow-up period (Month)	Mean (STD)	30.8035 (17.2336)	46.7595 (26.9758)	<0.0001
Death Event				<0.0001
	Death	37	14	
	Censored	580	56	

*STD* standard deviation.

The external validation cohort was collected from the Cancer Imaging Archive (TCIA) non-small cell lung cancer (NSCLC) Radiogenomics dataset^58–61^. We started with 211 patients and retained 70 patients based on the following inclusion criteria: the availability of a preoperative non-contrast CT, diagnosis of adenocarcinoma, and availability of survival outcome and demographic information. The imaging parameters for an external validation cohort were as follows: slice thickness of 0.625–3 mm (median of 1.5 mm), 80–140 kVp (mean 120 kVp), 124–699 mA (mean 220 mA).

### Tumor region of interest

Images in the training and test cohorts (from local data) were displayed in standard mediastinal (window width, 400 Hounsfield units (HU); window level, 20 HU) and lung (window width, 1500 HU; window level, −700 HU) windows. On serial axial CT images displayed at the lung window settings, the region of interest (ROI) of the whole tumor was segmented by two chest radiologists (HYL with 17 years of experience and GL with 13 years of experience) using a semi-automated process. Two sets of ROIs were obtained. ROIs were drawn on transverse CT scans at reconstruction intervals of 1 mm from the top to the bottom of the tumor, thus covering the entire tumor. The tumor margin including the ground glass component was defined as the ROI for the local cohort. The reproducibility of the ROIs and radiomics features were assessed using Cohen’s kappa and the ICC, respectively.

The tumor ROIs were not drawn on the external validation cohort because they were used to evaluate the DL model, not the radiomics model. The validation cohort was used to see if the developed radiomics-guided DL model showed reasonable performance on the independent cohort.

### Construction of baseline radiomics model for prognosis

Seventy-two radiomics features were computed using the open-source software PyRadiomics^49^, and six additional marginal features from our previous study^50^ were calculated using an in-house code implemented in MATLAB (MathWorks, Natick, MA, USA) from the first set of ROIs. A total of 78 radiomics features were calculated reflecting the shape, intensity distribution, texture (GLCM and GLSZM), and margin characteristics (slope of CDF of tumor intensity). Each feature was z-score normalized based on the mean and standard deviation of the feature values of the training cohort.

We adopted the Cox model regularized by the least absolute shrinkage and selection operator (Cox-LASSO) in the training cohort to select radiomics features to build the RRS model. The RRS integrates selected radiomics features to predict patient risk and this term has been used in the previous studies^14^. Optimal coefficients were found by nested 5-fold cross-validation and grid search process. The RRS was defined as a relative risk at the initial time according to the following equation:

1 $${RR}{S}_{i}=h\left({X}_{i},0\right)={h}_{0}(0)\cdot {{{{{{\rm{e}}}}}}}^{\mathop{\sum }\limits_{j=1}^{n}{x}_{{ij}}* {\beta }_{j}},$$

where $h\left({X}_{i},0\right)$ denotes the initial hazard of the *i*th patient whose feature vector is ${X}_{i}$, ${x}_{{ij}}$ denotes *j*th the prognostic feature of the *i*th patient, *n* denotes the number of selected features, and ${\beta }_{j}$ denotes the corresponding Cox-LASSO coefficient of the *j*th feature. ${h}_{0}(0)$ is the constant output of the shared hazard function at the initial time point. The same RRS model was applied to the test cohort fixing the model parameters and using the feature values from the test cohort to obtain the RRS for the test cohort.

Risk groups were stratified by applying the median RRS score of the training cohort to both the training and test cohorts. Individuals whose risk scores were the exact median were assigned to the low-risk group. Survival analysis was conducted using a Kaplan–Meier plot and log-rank tests. The HR, *p*-value, and C-index were measured.

### Construction of radiomics-guided pretrained DL model for prognosis

Figure 1b shows a graphical description of selecting DL features guided by radiomics for prognosis. CT images were cropped into 128x128x3-sized patches whose center slice showed the largest spatial extent of the tumor in 2D. To extract abstract high-level information of lung adenocarcinomas, the image patches were fed to the pretrained You Only Look Once (Yolo) v3^62^, Dense^63^, and VGG^64^ networks without optimization (i.e., frozen weight). The DL feature extraction was implemented using an open-source deep learning library, PyTorch^65^.

The Yolo v3 network consists of 107 convolutional layers and additional detection layers. We used the output of the 107th layer, the last layer before the detection layers, as the latent variables representing the DL features of the tumor patch. A 128 × 128 × 3-sized patch led to an output of 255 × 16 × 16 (=65,280) DL features. Additionally, we adopted 201-layered pretrained Dense and 19-layered pretrained VGG networks. The final classifier stages of the pretrained networks were removed so that the pre-trained networks could be used as feature extractors. Because the minimum size of the input images for these two networks was 144 × 144 × 3, the image patch used for Yolo v3 was upsampled to 256 × 256 × 3. The final classifiers were eliminated to be used as a feature extractor. As a result, 122,800 and 25,088 DL features were calculated for DenseNet and VGG, respectively.

All DL features were z-score transformed to the normalized range before conducting further processing. To filter the potential prognostic DL features, we calculated the correlation of all possible pairs of DL features and radiomics features and retained the DL features with absolute Pearson’s correlation of over 0.4 and Bonferroni-corrected *p*-value less than 0.05 in the training cohort. We focused on explaining a few important DL features in the final layer. Thus, we intended to build a compact prognostic DL model by correlating DL features with radiomics features. The chosen DL features were subjected to the same feature selection approach of Cox-LASSO used to build the DRS model as Eq. (1). The same DRS model was applied to the test and validation cohorts while fixing the model parameters and by using the feature values from the test and validation cohorts to obtain their DRS. The risk groups were stratified by applying the median DRS score of the training cohort to the training, test, and validation cohorts.

### Statistics and reproducibility

We used two-sample Student’s *t* tests to compare continuous-valued information and Chi-square tests to compare categorized information in the demographic table. To compare the risk groups, a log-rank test was adopted. All statistical analyses were conducted using the Statistics and Machine Learning Toolbox in MATLAB. Our code and model are available on GitHub and Zenodo (https://github.com/Hwan-ho/RGDL)^66^. The reproducibility of radiomics features was measured by calculating the ICC. Results of the survival analysis were validated with open-source dataset (TCIA-Radiogenomics).

### Reporting summary

Further information on research design is available in the Nature Research Reporting Summary linked to this article.

## Supplementary information

Supplementary Material

Description of Additional Supplementary Files

Supplementary Data 1

Supplementary Data 2

Supplementary Data 3

Reporting Summary

## Data Availability

Data of the external validation cohort are from The Cancer Imaging Archive (i.e., TCIA-Radiogenomics) and they are open to the public (https://wiki.cancerimagingarchive.net/display/Public/NSCLC+Radiogenomics). Data of the local cohort are from the Samsung Medical Center (SMC) and available with permission from the IRB of SMC. The permission is necessary due to the approved IRB restriction.

## Code availability

Our software code is open (https://github.com/Hwan-ho/RGDL) with the following (10.5281/zenodo.5543280)^66^.
