# Supplementary material for: Radiomics-guided deep neural networks stratify lung adenocarcinoma prognosis from CT scans
Source: Commun Biol. 2021 Nov 12;4:1286. doi: 10.1038/s42003-021-02814-7 (PMC8590002; doi:10.1038/s42003-021-02814-7)
Supplement: Supplementary file 1 — Supplementary Material [file 42003_2021_2814_MOESM1_ESM.pdf]

Supplementary Figures

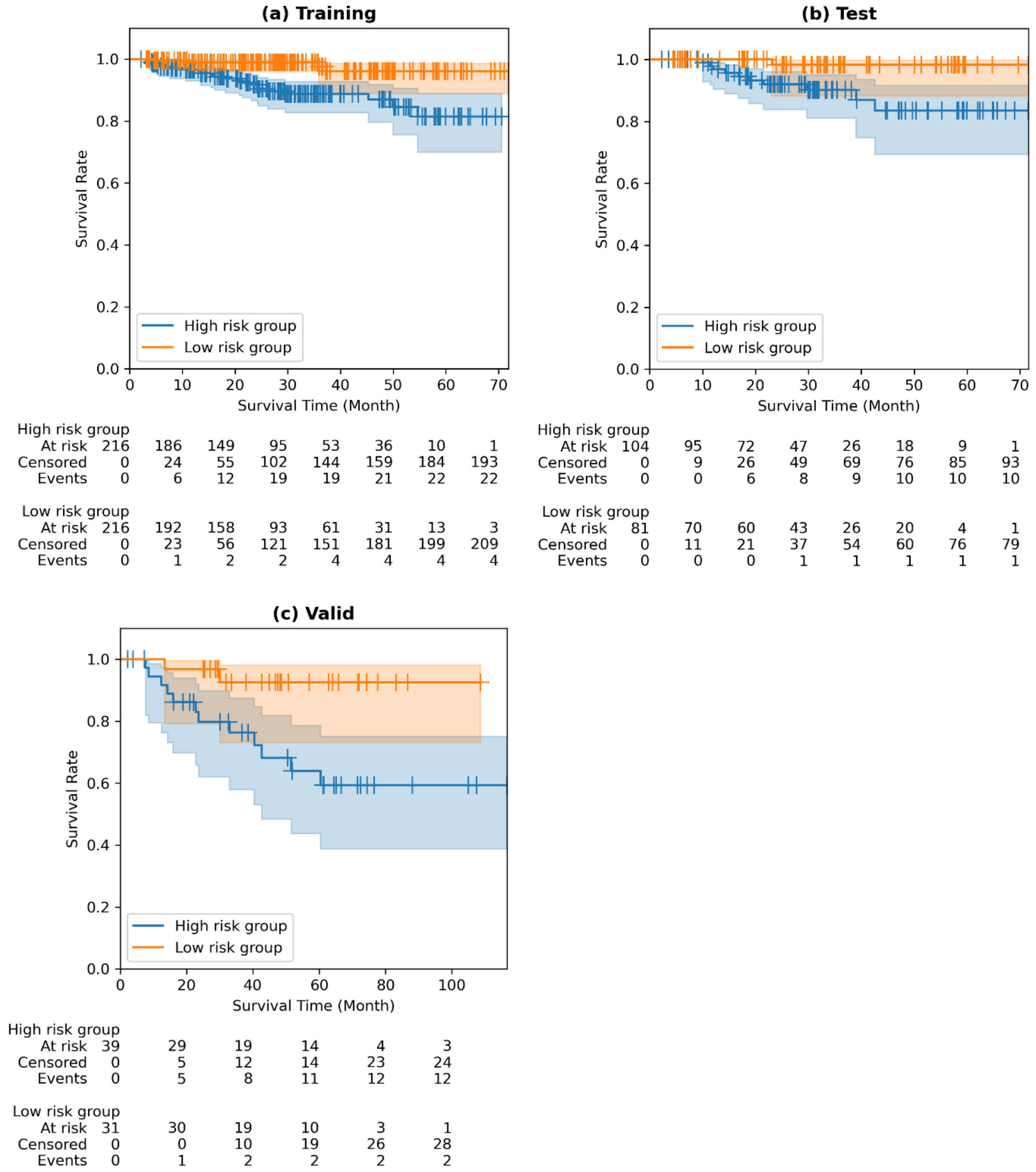

Figure S1. Kaplan–Meier plots of radiomics-guided DL model using Dense network. (a) Kaplan–Meier plot of training cohort, hazard ratio of 4.4383 (95% CI of 2.0468–9.6241), p-value of 0.0003, and C-index of 0.7410, (b) Kaplan–Meier plot of test cohort, hazard ratio of 5.3620 (95% CI of 1.7011–18.5774), p-value of 0.0116, and C-index of 0.0.7214, (c) Kaplan–Meier plot of validation cohort, hazard ratio of 4.2277 (95% CI of 1.4800–12.0768), p-value of 0.0153, and C-index of 0.7112.

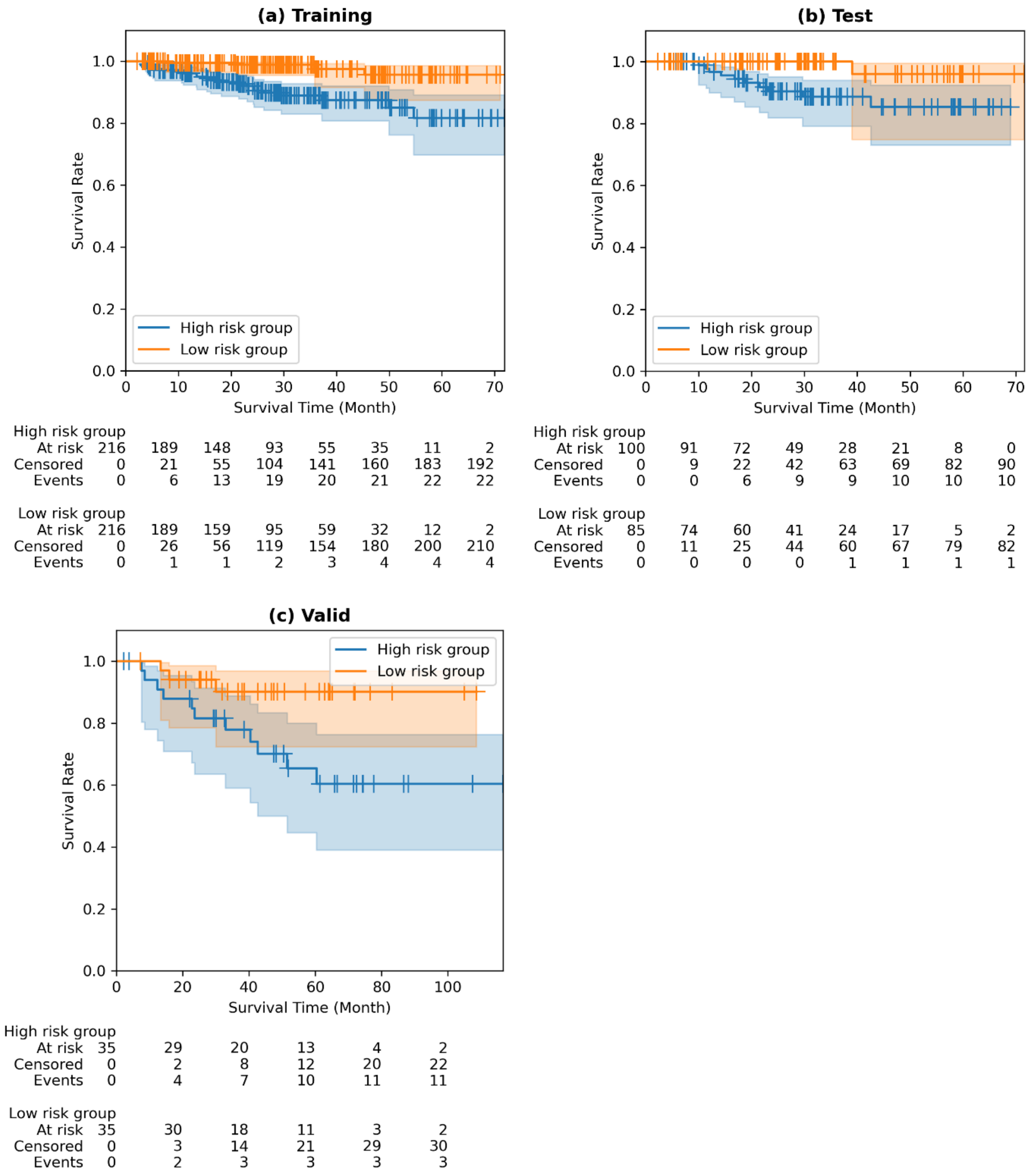

Figure S2. Kaplan–Meier plots of radiomics-guided DL model using VGG network. (a) Kaplan–Meier plot of training cohort, hazard ratio of 4.2454 (95% CI of 1.9658–9.1683), p-value of 0.0005, and C-index of 0.7660, (b) Kaplan–Meier plot of test cohort, hazard ratio of 4.7060 (95% CI of 1.4333–15.4520), p-value of 0.0244, and C-index of 0.0.8009, (c) Kaplan–Meier plot of validation cohort, hazard ratio of 3.5488 (95% CI of 1.2409–10.1494), p-value of 0.0362, and C-index of 0.6757.

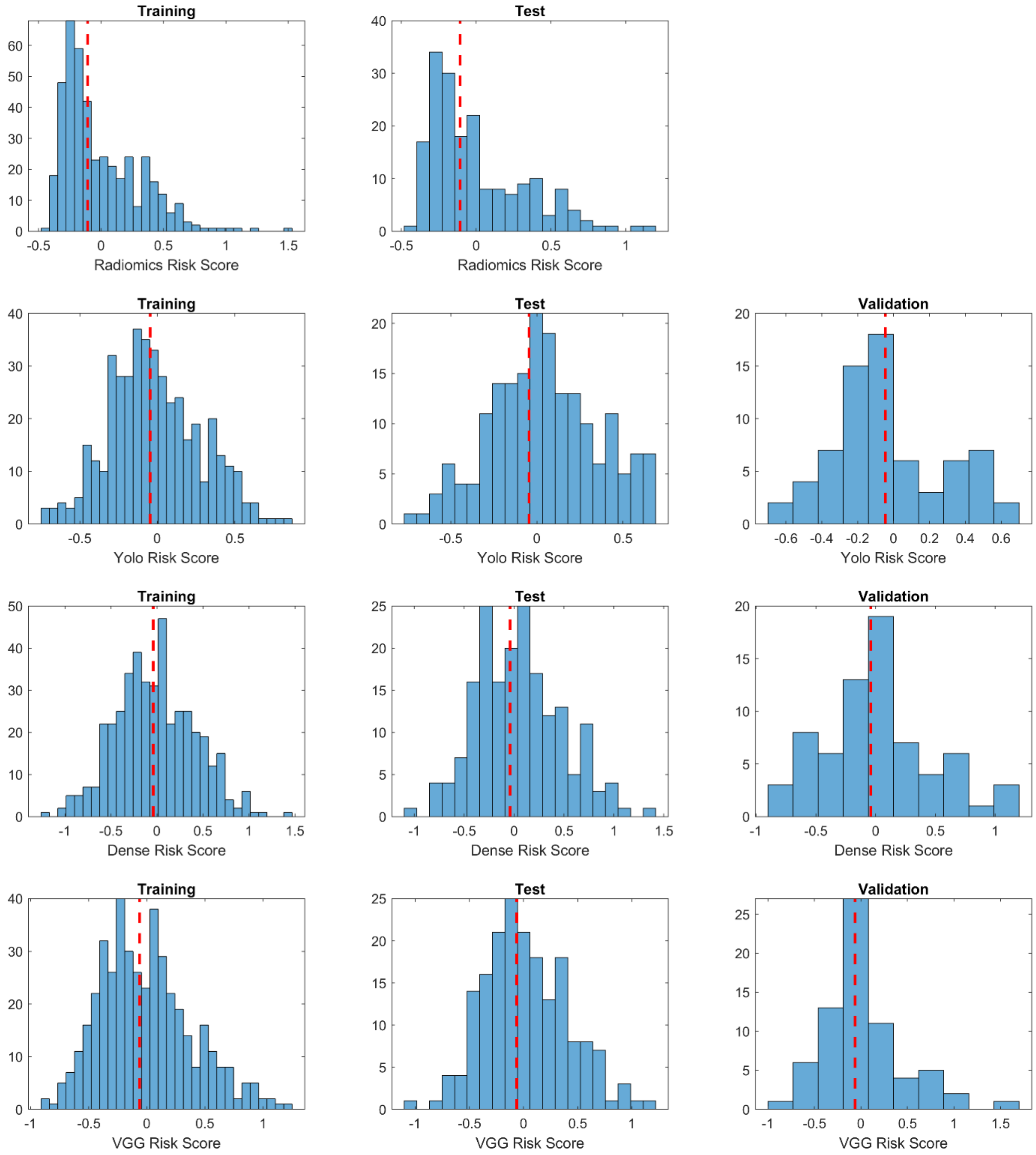

Figure S3. Plot of RRS and DRS for various approaches. The red dotted line is the median cut-off.

Supplementary Tables

Table S1. DL risk score model using Dense network for prognosis and the associated DL features.

Feature	Cox-LASSO coefficient
Dense_Latent_5092	0.0752
Dense_Latent_23965	0.1629
Dense_Latent_34596	-0.1983
Dense_Latent_34597	-0.0995
Dense_Latent_49309	0.1584

Table S2. DL risk score model using VGG network for prognosis and the associated DL features.

Feature	Cox-LASSO coefficient
VGG_Latent_9481	-0.0763
VGG_Latent_9482	-0.2061
VGG_Latent_21151	0.1891
VGG_Latent_23055	0.0385
VGG_Latent_25064	-0.0349

Table S3. Performance of various radiomics-guided DL models using features extracted from intermediate layers. We used the 80th, 150th, and 15th layers from the last layer for the Yolo, DenseNet201, and VGG19 networks.

	**Training**			
	**Hazard ratio**	**95% CI**	**p-value**	**C-index**
**Yolo**	4.4436	2.0300 - 9.7268	0.0004	0.7271
**DenseNet**	2.8972	1.3376 - 6.2754	0.0124	0.7250
**VGG**	3.8206	1.7645 - 8.2726	0.0014	0.7226
	**Test**			
	**Hazard ratio**	**95% CI**	**p-value**	**C-index**
**Yolo**	2.1897	0.6585 - 7.2817	0.3311	0.6492
**DenseNet**	2.6842	0.8815 - 8.8788	0.1893	0.6798
**VGG**	3.3727	1.0250 - 11.0974	0.0897	0.7236
	**Validation**			
	**Hazard ratio**	**95% CI**	**p-value**	**C-index**
**Yolo**	1.6041	0.5500 - 4.6780	0.5537	0.5192
**DenseNet**	2.7760	0.9703 - 7.9416	0.1019	0.7269
**VGG**	2.1097	0.7367 - 6.0418	0.2617	0.6294
