# Supplementary material for: Radiomics-guided deep neural networks stratify lung adenocarcinoma prognosis from CT scans
Source: Commun Biol. 2021 Nov 12;4:1286. doi: 10.1038/s42003-021-02814-7 (PMC8590002; doi:10.1038/s42003-021-02814-7)
Supplement: Supplementary file 2 — Description of Additional Supplementary Files [file 42003_2021_2814_MOESM2_ESM.pdf]

Description of Additional Supplementary Files

File name: Supplementary Data 1.
Description: ICC values for the radiomics features.

File name: Supplementary Data 2.
Description: Source data for Figure 4.

File name: Supplementary Data 3.
Description: Source data for all Kaplan-Meier plots.
