# Supplementary material for: Radiomics-guided deep neural networks stratify lung adenocarcinoma prognosis from CT scans
Source: Commun Biol. 2021 Nov 12;4:1286. doi: 10.1038/s42003-021-02814-7 (PMC8590002; doi:10.1038/s42003-021-02814-7)
Supplement: Supplementary file 6 — Reporting Summary [file 42003_2021_2814_MOESM6_ESM.pdf]

Corresponding author(s): Hyunjin Park

Last updated by author(s): Mar 30, 2021

Reporting Summary

Nature Research wishes to improve the reproducibility of the work that we publish. This form provides structure for consistency and transparency in reporting. For further information on Nature Research policies, see our Editorial Policies and the Editorial Policy Checklist.

Statistics

For all statistical analyses, confirm that the following items are present in the figure legend, table legend, main text, or Methods section.

n/a	Confirmed	
☐	☒	The exact sample size (n) for each experimental group/condition, given as a discrete number and unit of measurement
☐	☒	A statement on whether measurements were taken from distinct samples or whether the same sample was measured repeatedly
☐	☒	The statistical test(s) used AND whether they are one- or two-sided<br>*Only common tests should be described solely by name; describe more complex techniques in the Methods section.*
☐	☒	A description of all covariates tested
☐	☒	A description of any assumptions or corrections, such as tests of normality and adjustment for multiple comparisons
☐	☒	A full description of the statistical parameters including central tendency (e.g. means) or other basic estimates (e.g. regression coefficient) AND variation (e.g. standard deviation) or associated estimates of uncertainty (e.g. confidence intervals)
☐	☒	For null hypothesis testing, the test statistic (e.g. F, t, r) with confidence intervals, effect sizes, degrees of freedom and P value noted<br>*Give P values as exact values whenever suitable.*
☒	☐	For Bayesian analysis, information on the choice of priors and Markov chain Monte Carlo settings
☒	☐	For hierarchical and complex designs, identification of the appropriate level for tests and full reporting of outcomes
☒	☐	Estimates of effect sizes (e.g. Cohen's d, Pearson's r), indicating how they were calculated

Our web collection on statistics for biologists contains articles on many of the points above.

Software and code

Policy information about availability of computer code

Data collection	n/a
Data analysis	PyRadiomics, Yolo v3, python 3.6, MATLAB 2019b

For manuscripts utilizing custom algorithms or software that are central to the research but not yet described in published literature, software must be made available to editors and reviewers. We strongly encourage code deposition in a community repository (e.g. GitHub). See the Nature Research guidelines for submitting code & software for further information.

Data

Policy information about availability of data

All manuscripts must include a data availability statement. This statement should provide the following information, where applicable:

- Accession codes, unique identifiers, or web links for publicly available datasets
- A list of figures that have associated raw data
- A description of any restrictions on data availability

The full imaging and phenotypic data from The Cancer Imaging Archive (TCIA) are available after approval (https://www.cancerimagingarchive.net/).
Our software code will be provided through gitHub repository (https://github.com/Hwan-ho/RGDL).

Field-specific reporting

Please select the one below that is the best fit for your research. If you are not sure, read the appropriate sections before making your selection.

☒ Life sciences ☐ Behavioural & social sciences ☐ Ecological, evolutionary & environmental sciences

For a reference copy of the document with all sections, see nature.com/documents/nr-reporting-summary-flat.pdf

Life sciences study design

All studies must disclose on these points even when the disclosure is negative.

Sample size	Local cohort: 617; External validation cohort: 70
Data exclusions	Local cohort: Patient with a history of previous radiation or chemotherapy were excluded.<br>External validation cohort: Patients with contrast enhanced CT, patients who were not diagnosis of adenocarcinoma, and patients whose survival outcome and demographic information were not available were excluded.
Replication	For regions of interest (ROI) reproducibility, we measured intra-class correlation (ICC) of radiomics features and Cohen's kappa using ROI segmented by two radiologists. Both ROI and radiomics feature were confirm to be reproducible with high agreement.<br>The deep learning risk score model built with proposed methodology using local cohort was validated with external validation cohort.
Randomization	For risk score model building, the local cohort was randomly separated into a7:3 ratio maintaining demographical distribution and used as training and test cohorts.
Blinding	NA

Reporting for specific materials, systems and methods

We require information from authors about some types of materials, experimental systems and methods used in many studies. Here, indicate whether each material, system or method listed is relevant to your study. If you are not sure if a list item applies to your research, read the appropriate section before selecting a response.

Materials & experimental systems

n/a	Involved in the study	
☒	☐	Antibodies
☒	☐	Eukaryotic cell lines
☒	☐	Palaeontology and archaeology
☒	☐	Animals and other organisms
☐	☒	Human research participants
☐	☒	Clinical data
☒	☐	Dual use research of concern

Methods

n/a	Involved in the study	
☒	☐	ChIP-seq
☒	☐	Flow cytometry
☒	☐	MRI-based neuroimaging

Human research participants

Policy information about studies involving human research participants

Population characteristics	Local cohort: age (60.22, SD 9.37), Sex (43% male), Smoke history (38% Yes), Follow up period (30.80 month, SD 17.23), Death event (6% Death)<br>External validation cohort: age (66.97, SD 11.18), Sex (56% male), Smoke history (71% Yes), Follow up period (46.76 month, SD 26.98), Death event (20% Death)
Recruitment	Local cohort: Obtained from Samsung Medical Center, Seoul, Korea<br>External validation cohort: Obtained from publicly accessible databases
Ethics oversight	Local cohort: The Institutional Review Board of Samsung Medical Center approved the prospective data analysis.<br>External validation corhot: The Institutional Review Board approval is waived for the analysis of retrospective data.

Note that full information on the approval of the study protocol must also be provided in the manuscript.

Clinical data

Policy information about clinical studies

All manuscripts should comply with the ICMJE guidelines for publication of clinical research and a completed CONSORT checklist must be included with all submissions.

Clinical trial registration	NCT01482585
Study protocol	*Note where the full trial protocol can be accessed OR if not available, explain why.*
Data collection	*Describe the settings and locales of data collection, noting the time periods of recruitment and data collection.*
Outcomes	*Describe how you pre-defined primary and secondary outcome measures and how you assessed these measures.*
